# Prognostic Power of Pathogen Cell-Free DNA in *Staphylococcus aureus* Bacteremia

**DOI:** 10.1093/ofid/ofz126

**Published:** 2019-03-15

**Authors:** Alessander O Guimaraes, Johnny Gutierrez, Stacey A Maskarinec, Yi Cao, Kyu Hong, Felicia Ruffin, Montserrat Carrasco-Triguero, Melicent C Peck, Vance G Fowler, Amos Baruch, Carrie M Rosenberger

**Affiliations:** 1 Biomarker Discovery, Genentech, Inc., South San Francisco, California; 2 Bioinformatics and Computational Biology, Genentech, Inc., South San Francisco, California; 3 Biomarker Development, Genentech, Inc., South San Francisco, California; 4 Division of Infectious Diseases, Duke University Division of Infectious Diseases, Durham, North Carolina; 5 Bioanalytical Sciences, Genentech, Inc., South San Francisco, California; 6 Clinical Sciences, Genentech, Inc., South San Francisco, California

**Keywords:** bacteremia, cell-free DNA, prognostic biomarkers, *Staphylococcus aureus*

## Abstract

**Background:**

*Staphylococcus aureus* is a leading global cause of bacteremia that can cause invasive tissue infections with high morbidity and mortality despite appropriate antibiotic therapy. Clinicians lack sufficient tools to rapidly identify patients with a poor prognosis to guide diagnostic workup and treatment decisions. Host cell-free DNA provides prognostic value across a spectrum of critical illnesses, including *S. aureus* bacteremia and sepsis. Metrics of high bacterial load are associated with disease severity in *S. aureus* bacteremia, and the objective of this study was to evaluate whether incorporating quantitation of cell-free bacterial DNA would provide additive prognostic value when combined with biomarkers of the inflammatory response.

**Methods:**

*S. aureus* cell-free DNA was measured by quantitative polymerase chain reaction (PCR) in baseline serum samples from an observational cohort of 111 patients with complicated *S. aureus* bacteremia and correlated with host inflammatory markers and clinical outcomes.

**Results:**

High levels of *S. aureus* cell-free DNA at the time of positive index blood culture were prognostic for all-cause and attributable mortality and persistent bacteremia and were associated with infective endocarditis. However, they did not provide additive value to biomarkers of the host response to infection in multivariate analysis.

**Conclusions:**

Measurements of bacterial load by PCR are a clinically feasible candidate biomarker for stratifying patients at higher risk for complications and poor outcomes. Their diagnostic and prognostic value for identifying foci of infection and influencing treatment remain to be evaluated in additional cohorts.

*Staphylococcus aureus* is a leading cause of bacteremia worldwide. Mortality and complications such as persistent bacteremia and infection relapse have been observed in >30% of cases despite appropriate antibiotic therapy, and patients with complicated tissue foci of infections are more likely to have poor outcomes [1, 2]. Risk stratification biomarkers offer a potential adjunctive tool for the identification of patients with *S. aureus* bacteremia at higher risk for poor outcomes, which could not only inform best management practices, but also facilitate clinical development of new drugs by identifying the patients who would most benefit from novel therapeutics [3].

Prior studies have demonstrated that high circulating levels of human cell-free DNA at admission are prognostic for sepsis and mortality in patients with *S. aureus* infections, highlighting the potential of this type of biomarker in clinical risk stratification [4–6]. Extracellular DNA can be released following cell death or by an active cellular process induced by tissue injury, which is subsequently detected in membrane-bound vesicles or exosomes and can be stably measured in blood (reviewed in [7]). Extracellular DNA has been proposed as a surrogate marker for the extent of underlying tissue damage, and can also directly augment disease severity by promoting additional inflammation through the activation of TLR9 or intracellular DNA receptors after uptake by phagocytes such as neutrophils (reviewed in [8]) and coagulation ([9], reviewed in [10]). Both nuclear and mitochondrial DNA have been implicated in the inflammatory host response to pathogens and tissue injury such as that seen in sepsis and have been linked to worse outcomes in intensive care unit patients [11–14].

The prognostic value of cell-free *S. aureus* DNA has not previously been evaluated, although metrics of higher *S. aureus* bacterial load such as blood colony-forming unit (CFU), time to blood culture positivity, and endovascular inoculum have been associated with bacteremia persistence and mortality in patients with *S. aureus* bacteremia [15–19]. In this study, we sought to evaluate the usefulness of quantifying bacterial cell-free DNA for identifying the subset of patients with complicated *S. aureus* bacteremia who are more likely to have worse outcomes. We hypothesized that measuring bacterial cfDNA may provide a more direct measure of pathogen burden than downstream host factors [15, 20–24]. We assessed the relationships between bacterial cell-free DNA and disease severity, with a particular interest in the clinical outcomes most closely linked to the causative pathogen: persistent bacteremia and infection-attributable mortality. We previously identified 3 serum cytokines associated with mortality (IL-8 and CCL-2) or persistent bacteremia (IL-17A) in patients with complicated *S. aureus* bacteremia that were prognostically superior to routinely available clinical metrics such as blood cellularity or clinical chemistry [20]. We evaluate the prognostic value of baseline bacterial cell-free DNA alone and combined with prognostic host inflammatory metrics for mortality and persistent bacteremia.

## METHODS

### Experimental Design

We isolated cell-free circulating DNA from serum samples available from 111 of 124 patients in a previously reported retrospective observational study designed to evaluate the prognostic value of serum biomarkers for disease severity outcomes in patients with *S. aureus* bacteremia [20]. The cohort was composed of subjects with serum samples collected within 1–3 days of the date of the index blood culture from a Duke University (Durham, NC, USA) sample repository of patients with confirmed complicated *S. aureus* bacteremia based on the 2015 Infectious Diseases Society of America (IDSA) criteria [25], collected under protocols approved by the institutional ethics review boards (IRB protocol Pro00008031). The cohort was selected to enrich for persistent bacteremia and mortality in addition to a control group of patients with complicated *S. aureus* bacteremia enrolled over the same time period matched for demographic variables and infection source [20]. Demographic and clinical characteristics are shown in Table 1 and Supplementary Table 1. Serum cell-free circulating DNA from 16 healthy volunteers was used as a control for nuclear and mitochondrial cell-free DNA quantification.

**Table 1.** Characteristics of Clinical Study

No. of patients	111
Age, median (range), y	62 (22–91)
Female, No. (%)	49 (44.1)
Outcome, No. (%)	
Mortality	25 (22.5)
Attributable mortality	19 (17.1)
Persistent bacteremia	53 (47.7)
Complicated infection	111 (100)
Recurrence	14 (12.6)
LOS, median (range), d^a^	22 (4–122)
Treatment duration, median (range), d	39 (2–113)
MRSA, No. (%)	66 (59.5)
Infection foci, No. (%)	
Endovascular	60 (54.1)
Extravascular osteoarticular	20 (18.0)
Extravascular soft tissue	7 (6.3)
Other	24 (21.6)
Comorbidities, No. (%)	
Diabetes	52 (46.8)
Hemodialysis dependent	43 (38.7)
Cancer	19 (17.1)
Transplant recipient	10 (9.0)
HIV+	4 (3.6)

Abbreviations: LOS, length of stay; MRSA, methicillin resistant *Staphylococcus aureus.*

^a^Fatal cases and patients who left the hospital against medical advice were excluded from hospital length of stay. Patients with 2 or more identified foci of infection were assigned hierarchically first to “endovascular,” second to “extravascular osteoarticular,” third to “extravascular soft tissue,” and finally to “other,” which included catheter-associated infections and unidentified foci of infection. Age of healthy control cohort: median (range), 50 (32–66) years.

### Clinical Severity Definitions

All-cause mortality was defined death during hospitalization or up to 90 days from the index positive blood culture, and attributable mortality was assessed retrospectively by a board-certified infectious disease physician based on review of patient charts. Persistent bacteremia was defined as a documented positive blood culture for *S. aureus* for ≥5 days. Duration of positive blood culture was calculated as days to last positive blood culture.

Patients were subdivided into 4 categories regarding foci of infection: endovascular, when diagnosis or clinical evidence was present for infective endocarditis, septic thrombophlebitis, septic emboli, hemodialysis graft infection, pacemaker or any cardiovascular medical device infection; osteoarticular, when not classified as endovascular and with diagnosed osteomyelitis, joint infection, septic arthritis, and orthopedic or prosthetic device implant infections; extravascular soft tissue abscess, with a diagnosis of cellulitis, meningitis, fasciitis, empyema, pneumonia, calcified infarct infection, soft tissue or skin abscess; other, which included complicated catheter-related infections and any patients with an unknown foci of infection. For biomarker associations with infection foci categories, patients were assigned in a hierarchical manner when 2 or more foci were identified (endovascular > osteoarticular > soft tissue > other); for example, patients with both endovascular and osteoarticular infections were assigned to the endovascular category.

### Biomarker Measurements

#### Serum Cell-Free DNA Isolation

Total serum cell-free DNA (host and bacterial) was obtained using the methodology reported in Gutierrez et al. [26]. To exclude measuring DNA from intact bacteria, serum samples of 150 μL were previously centrifuged at 16 000*g* for 5 minutes, and the top 100 μL was transferred to MagNA Pure Compact (Roche) sample tubes for automated DNA extraction, per manufacturer instructions.

#### S. aureus *Cell-Free DNA Quantitative Polymerase Chain Reaction*

As described in Gutierrez et al., [26] custom primers and TaqMan probe were designed to an alignment of the 16S rRNA gene region specific to *Staphylococcus*—16S-01-FP (5’-CCGCATGGTTCAAAAGTGAAA-3’), 16S-01-RP (5’-GCAGCGCGGATCCATCTA T-3’, 16S-R-FAM-NFQ (ACGGTCTTGCTGTCACT)—and were used in quantitative polymerase chain reaction (PCR) carried out on the purified sample DNA and known copy number of DNA standard. The 16S rRNA gene copy number can vary from 5 to 6 copies per bacterium, so measurements reflect total genome copies rather than an absolute measurement of bacterial load. The assay limit of quantification (LLOQ) was 50 copies 16S rRNA copies/mL, and the 45% of patients who had undetectable or below-LLOQ results were assigned the LLOQ value for analysis. Detailed procedures can be found in the Supplementary Data.

#### Cytokines

Serum proteins were measured using the Ella platform (ProteinSimple, San Jose, CA) or Luminex platform (R&D Systems, Minneapolis, MN) as previously described [20].

### Statistical Analysis

Mann-Whitney tests, Spearman’s rank correlations, and receiver operating characteristics (ROC) curves were performed using GraphPad Prism (version 7.0d for MAc OS X; GraphPad Software, La Jolla, CA; www.graphpad.com). Logistic regression models combining SAcfDNA, age, CCL-2, IL-8, and IL-17, as described in the “Results,” were obtained using glm in R [27]. Biomarkers and clinical variable levels that are significantly different between the outcome groups were identified using the Wilcoxon rank-sum test, and nonparametric Spearman’s rank correlation coefficients were calculated to determine the relationship between biomarker levels. Illustrative cutoffs were defined by calculating Youden’s J for the maximal combined sensitivity and specificity.

## RESULTS

### *S. aureus* Cell-Free DNA Is Prognostic for Mortality

We measured circulating levels of *S. aureus* cell-free DNA (SAcfDNA) in serum samples from a previously reported case–control observational cohort of patients with *S. aureus* bacteremia designed to investigate the prognostic power of biomarker candidates for mortality and persistent bacteremia (cohort characteristics in Table 1) [20]. Serum samples were collected within 1–3 days of the date of the index positive blood culture for *S. aureus*. SAcfDNA was quantifiable in 55% of the patients and had prognostic value for 90-day all-cause mortality and persistent bacteremia (positive blood cultures for ≥5 days) (Table 2).

**Table 2.** Prognostic Power of *S. aureus* Cell-Free DNA vs Age and APACHE II Score at Enrollment for All-Cause Mortality, Attributable Mortality, and Persistent Bacteremia

	Mortality		Infection-Related Mortality		Persistent Bacteremia	
	*P*	AUROC	*P*	AUROC	*P*	AUROC
SAcfDNA	.0055^**^	0.67	.0007^***^	0.73	.0045^**^	0.65
Age	.0075^**^	0.67	.032*	0.66	.52	
APACHE II	.039*	0.64	.0082^**^	0.69	.22	

APACHE II score and persistent bacteremia = positive blood culture ≥5 days on appropriate antibiotics. *P* values from Mann-Whitney test (*P < .05; ^**^P < .01; ^***^P < .001).

Abbreviations: AUROC, area under the receiver operating characteristics curve; SAcfDNA, circulating *Staphylococcus aureus* cell-free DNA.

**Figure 1.**
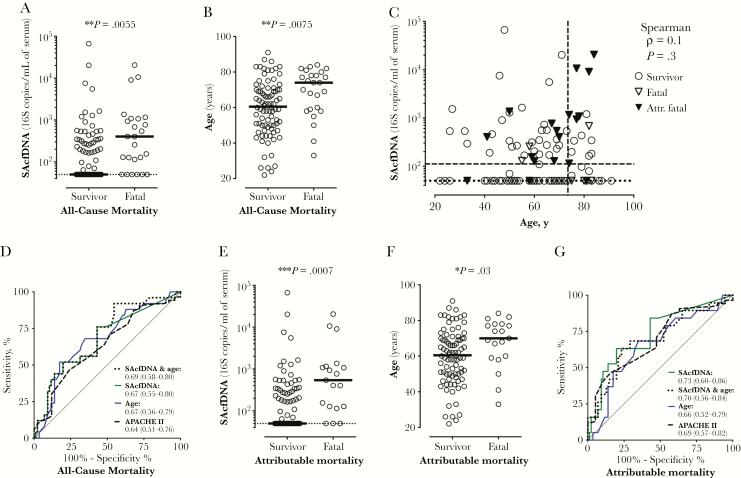
*Staphylococcus aureus* cell-free DNA level is prognostic for mortality. A, *S. aureus* cell-free DNA (SAcfDNA) at enrollment and (B) age were significantly higher in fatal (n = 25) vs survivor (n = 86) cases. C, Lack of Spearman correlation between SAcfDNA and age. Dashed lines indicate cutoffs, defined by maximum combined sensitivity and specificity for classifying fatal outcome. Dotted lines = polymerase chain reaction lower limit of quanification (LLOQ). D, Area under the receiver operating characteristics curve (AUROC) for the SAcfDNA and age logistic regression model compared with SAcfDNA, age alone, or APACHE II score in predicting all-cause mortality. E, A greater difference was observed in *S. aureus* cell-free DNA (SAcfDNA) at enrollment between attributable mortality (n = 19) and survivors (n = 86) than for (F) age. G, AUROC for the SAcfDNA and age logistic regression model compared with SAcfDNA, age alone, or APACHE II score in predicting attributable mortality. The legends of the receiver operating characteristics curves present their areas under the curve and respective 95% confidence intervals in parentheses. Wilcoxon rank-sum test *P* values and medians are shown; dotted lines indicate the LLOQ. *P < .05; ^**^P < .01; ^***^P < .001.

SAcfDNA at enrollment was significantly higher in patients who died within 90 days of positive diagnostic blood culture (median, 404 vs 50 copies/mL serum, the assay LLOQ for fatal [n = 25] vs survivors [n = 86]; *P* = .005) (Figure 1A). The only other parameters available at admission that were also associated with mortality were increasing age (median, 74 vs 61 years for fatal vs survivors; *P* = .007) (Figure 1B, Table 2; Supplementary Table 1) and clinical APACHE II score, which has age as 1 of the input parameters (APACHE II median, 17 vs 15 for fatal vs survival; *P* = .04) (Table 2; Supplementary Table 1). SAcfDNA did not correlate with age or with APACHE II scores (Figure 1C; Supplementary Table 2). We evaluated whether combining SAcfDNA and age would improve the ability to classify fatal outcome. To this end, we combined SAcfDNA and age in a logistic regression model that showed that both contributed significantly to distinguishing fatal from survival cases, albeit age had a lower contribution (SAcfDNA coefficient, 0.75; *P* = .02; age coefficient, 0.04; *P* = .002). However, the model did not predict mortality better than SAcfDNA or age alone, as indicated by similar area under the ROC (AUROC) curves (Figure 1D). Individual cutoff values discriminating mortality from survival were also calculated for SAcfDNA and age based on maximal combined specificity and sensitivity, which were age >73.5 years (52% sensitivity, 83% specificity) and SAcfDNA >111.5 copies/mL (76% sensitivity, 57% specificity). Based on these cutoffs, an additional 10 fatal cases were identified by age >73.5 years, and 4 cases were identified by SAcfDNA >111.5 copies/mL (Figure 1C), with a reduction in specificity.

SAcfDNA and age were also significantly higher in fatal cases attributed to infection (n = 19) compared with survivors (n = 86) (Figure 1E, F), which was also the case for the APACHE II score (median, 18 vs 15, attributable mortality [n = 19] vs survival [n = 86]; *P* = .008) (Table 2; Supplementary Table 1). Interestingly, SAcfDNA did have slightly better power to distinguish mortality from survival when considering only attributable mortality (AUROC, 0.73) rather than all-cause mortality (AUROC, 0.67), as did the APACHE II score (AUROC, 0.69 vs 0.64 for attributable and all-cause mortality, respectively), whereas age was equivalent (Figure 1D, G). In addition, there was no significant difference in median time to death between fatal cases attributed or not attributed to infection (attributable: median [range], 21 [7–87] days; n = 19; vs nonattributable: median [range], 19 [5–49] days; n = 6; *P* = .48). A logistic regression model combining age and SAcfDNA showed that only SAcfDNA significantly contributed to distinguishing cases of attributable mortality from survivors (SAcfDNA coefficient, 0.97; *P* = .006; age coefficient, 0.03; *P* = .08), and the model did not improve the prognostic value of SAcfDNA alone for attributable mortality (Figure 1G).

### *S. aureus* Cell-free DNA Is Prognostic for Persistent Bacteremia

**Figure 2.**
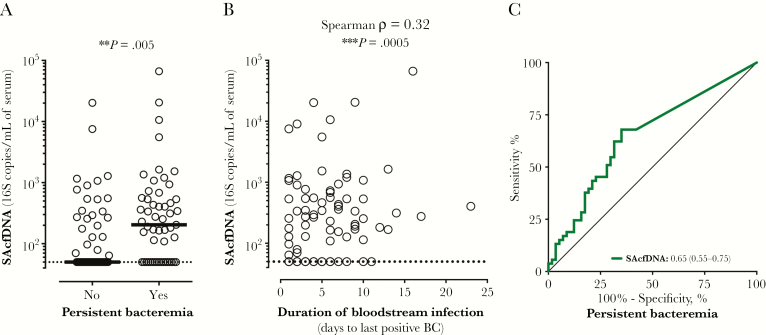
*Staphylococcus aureus* cell-free DNA (SAcfDNA) is prognostic value for discriminating persistent bacteremia. A, SAcfDNA was significantly higher at enrollment in the serum of patients with persistent (n = 53) vs resolving (n = 58) bacteremia; medians are shown, as well as Wilcoxon rank-sum test *P* value (^**^P < .01). B, Correlation between SAcfDNA and duration of positive blood cultures; Spearman’s *rho* and *P* value are (^***^P < .001) shown. C, SAcfDNA showed prognostic power to discriminate patients who developed persistent bacteremia, as evidenced by the significant area under the receiver operating characteristics curve shown in the legend, with 95% confidence interval in parentheses. Abbreviation: BC, blood culture.

SAcfDNA at enrollment was significantly higher in patients who subsequently developed persistent bacteremia (median, 204 vs 50 copies/mL, the assay LLOQ for persistent [n = 53] vs resolving bacteremia [n = 58]; *P* = .0001) (Figure 2A) and modestly correlated with bacteremia duration, as determined by blood culture positivity (Figure 2B). An SAcfDNA value at enrollment >103 copies/mL of serum discriminated between patients who did not have negative blood cultures within 5 days of the index positive blood culture with 68% sensitivity and 65% specificity (AUROC, 0.65; 95% confidence interval, 0.55–0.75) (Figure 2C). Age, cell counts, and creatinine did not differ significantly between patients with persistent and resolving bacteremia (Supplementary Table 1).

**Figure 3.**
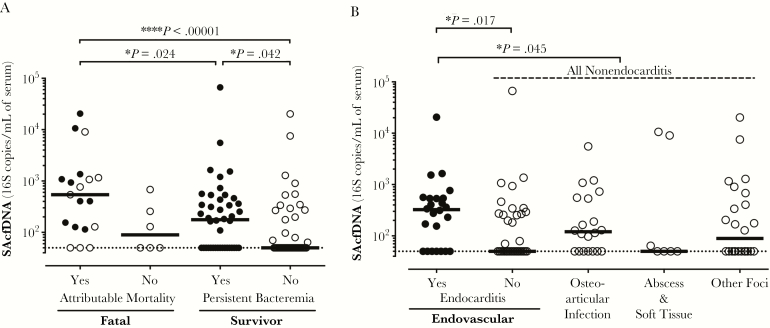
High-serum *Staphylococcus aureus* cell-free DNA (SAcfDNA) at enrollment in persistent bacteremia and infective endocarditis. A, Serum SAcfDNA at enrollment are highest in attributable mortality (n = 19), then survivors with persistent bacteremia (n = 42), then resolving bacteremia survivors (n = 44). Filled circles = persistent bacteremia. B, SAcfDNA was significantly higher in endocarditis patients (filled circles, n = 24) compared with all nonendocarditis cases (n = 87). SAcfDNA individual values for endovascular nonendocarditis (n = 36), osteoarticular infection (n = 20), soft tissue and abscess infections (n = 7), and other foci (n = 24) cases are shown. Medians and Wilcoxon rank-sum test *P* values (*P < .05; ^****^P < .0001) are presented; dotted lines indicate the lower limit of quanification.

As SAcfDNA is associated with both persistent bacteremia and mortality, we explored the inter-relationships between them. In addition to all-cause mortality (Figure 1A), differences in levels of SAcfDNA were seen between patients with attributable mortality and survivors with resolving bacteremia (541 vs 50 median copies/mL, the assay LLOQ; *P* < .00001) and survivors who did have persistent bacteremia (541 vs 176 median copies/mL; *P* = .02) (Figure 3A). This difference was driven by the fact that survivors with persistent bacteremia had a significantly higher level of SAcfDNA than survivors without persistent bacteremia (176 vs 50 median copies/mL; *P* = .042) (Figure 3A).

### *S. aureus* Cell-Free DNA Is Elevated in Patients With Infective Endocarditis

We examined relationships between SAcfDNA and infection foci. Patients with infective endocarditis presented with a significantly higher SAcfDNA when compared with other sources of *S. aureus* bacteremia (325 vs 64 median copies/mL; *P* = .045), and also higher endovascular sources (325 vs 50 median copies/mL of serum; *P* = .017) (Figure 3B). SAcfDNA was not significantly elevated when other sources of infection, such as osteoarticular, abscess, and soft tissue, were compared (Figure 3B). The recommended duration of antibiotic therapy for infective endocarditis is ≥6 weeks, and we observed an increased median level of cfDNA in patients with antibiotic treatment durations >6 weeks that was not statistically significant (Supplementary Figure 1A). Levels of SAcfDNA were not significantly different between infections caused by methicillin-resistant *S. aureus* vs methicillin-susceptible *S. aureus* (Supplementary Figure 1B). SAcfDNA was not prognostic for recurrence of *S. aureus* bacteremia, although this study was not designed to examine associations with this outcome.

The addition of *S. aureus* cell-free DNA to serum cytokines does not increase the prognostic power for mortality or persistent bacteremia.

**Figure 4.**
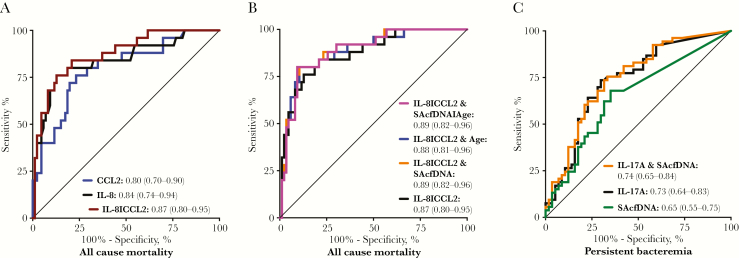
Combining *Staphylococcus aureus* cell-free DNA (SAcfDNA) with host inflammatory biomarkers did not improve the prognostic models for mortality and persistent bacteremia. A, Receiver operating characteristics (ROC) curves for IL-8, CCL-2, and a linear regression model combining these 2 analytes evidence a good prognostic power to distinguish all-cause mortality, as indicated by the respective under the receiver operating characteristic curve (AUROC). B, Incorporating SAcfDNA and/or age into the IL-8/CCL-2 linear regression model for all-cause mortality did not increase the AUROC significantly. C, A linear regression model combining SAcfDNA and IL-17A did not increase the AUROC over IL-17A alone. Legends in plots indicate respective ROCs and their AUROCs, followed by the 95% confidence interval in parentheses.

SAcfDNA did not correlate strongly with the 20 inflammatory cytokines previously examined in this cohort (Spearman correlations ≤ 0.4) (Supplementary Table 2). Modest correlations (Spearman ρ = 0.3–0.4) were observed between SAcfDNA and G-CSF, IL-6, and IL-17A (Supplementary Table 2), which suggest a link between SAcfDNA and neutrophilic inflammation. As SAcfDNA was not well correlated with IL-8 and CCL2, which is prognostic for all-cause mortality, nor with IL-17A, a cytokine prognostic for persistent bacteremia [20], we investigated whether combining SAcfDNA with these specific serum cytokines in logistic regression models would improve their prognostic power for all-cause mortality or persistent *S. aureus* bacteremia. IL-8, followed by CCL2, was the best prognostic analyte for mortality (Supplementary Table 1). As previously reported in the complete cohort [20], combining IL-8 and CCL2 values results in higher prognostic power for mortality, as indicated by a bigger AUROC for a logistic regression model combining these 2 cytokines than for each alone in the samples with the SAcfDNA measurements used in this study (Figure 4A). Logistic regression models combining SAcfDNA, IL-8, CCL2, and age did not show better prognostic value for all-cause mortality than the CCL2 with IL8 model (Figure 4B). Furthermore, SAcfDNA and age did not significantly contribute to distinguishing fatal cases from survivors when in combination with IL-8 and CCL2, as indicated by their nonsignificant small coefficients in this logistic regression model (Supplementary Table 3). When SAcfDNA was combined with IL-17A, the analyte previously reported as the sole biomarker prognostic power for distinguishing persistent bacteremia cases in this cohort [20], it did not improve the prognostic value of IL-17A by AUROC (Figure 4C) or in a logistic regression model (Supplementary Table 3).

## DISCUSSION

Biomarkers to drive risk-stratified diagnostic and treatment strategies are critically needed to improve outcomes in patients with complicated *S. aureus* bacteremia. In this study, we evaluated the prognostic utility of measuring circulating cell-free DNA levels within the first few days of positive index blood culture in patients with *S. aureus* bacteremia to identify patients at risk of poor outcomes. We observed that bacterial cell-free DNA was prognostic for all-cause and attributable mortality and persistent bacteremia in patients with complicated *S. aureus* bacteremia. SAcfDNA had a modestly stronger prognostic value for attributable mortality than age, an established risk factor for poor *S. aureus* bacteremia outcomes [2]. Although SAcfDNA was poorly correlated with age, the combination did not improve prediction of all-cause or attributable mortality. We measured higher quantities of SAcfDNA in the bloodstream of patients with persistent bacteremia and infective endocarditis. Persistent bacteremia is a characteristic feature of infective endocarditis [28]. We did not observe a relationship between high levels of SAcfDNA and other foci of infection. SAcfDNA identified patients at higher risk for persistent bacteremia, infective endocarditis, and mortality (both all-cause and attributable), better than age, APACHE II severity score, and the routine blood chemistry and cellularity metrics evaluated in this study.

Cell-free DNA is detectable in healthy individuals [7], and elevated levels are seen in a variety of pathological conditions, including infection, sepsis, trauma, and autoimmune diseases (reviewed in [10]). DNA release from bacteria can occur by antibiotic- or immune-mediated cell death and has been postulated to contribute to pathogenesis by promoting biofilm formation [29]. Bacterial DNA is one of many potential drivers of inflammation, which include other pathogen components such as peptidoglycan and host factors such as damage-associated molecular patterns and alarmins. Several recent papers with approaches ranging from quantifying cell-free DNA to understanding the encoded genetic and epigenetic information support the value of human cell-free DNA for diagnosis [30] and predicting mortality [4–6], with the potential to guide treatment decisions [31]. Bacterial cell-free DNA is also being evaluated as a diagnostic tool [32].

Few studies have combined host and bacterial metrics to assess their relative contributions to poorer clinical outcomes. Bacterial load can be difficult to quantify in *S. aureus* bacteremia due to inability to measure the burden of infection in tissue reservoirs and the rapid resolution of bacteremia in the presence of antibiotics. A few studies have demonstrated a relationship between bacterial load and poor clinical outcomes. Rose et al. [15] established a link between high viable bacterial burden in blood, higher levels of serum IL-10, and mortality in *S. aureus* bacteremia. Time to blood culture positivity (TTP) is considered a semiquantitative metric of blood bacterial load due to a variety of factors, including presence of antibiotics and volume of blood, with faster time to positive blood culture presumed to reflect a higher bacterial load. TTP has been associated with all-cause mortality [16–19], attributable mortality [18], persistent bacteremia (≥3 days) on appropriate antibiotics [18, 33], and infective endocarditis [17, 18, 34].

We previously reported on the potential clinical utility of serum cytokines in this cohort [20] and demonstrated that IL-8 and CCL2 in combination were prognostic for all-cause mortality and that IL-17A alone had better power to identify patients who developed persistent bacteremia than available clinical and lab metrics. SAcfDNA had a lower prognostic power for mortality or persistent bacteremia than these cytokines and did not improve the prognostic power of the cytokine-based prognostic models in multivariate analysis (Figure 4). We did not measure strong correlations between SAcfDNA and IL-10 or other cytokines previously associated with poor outcomes [15, 20–24]. The stronger correlation between inflammatory cytokines and viable bacterial load [15] compared with the single PAMP measured in this study suggests that a combination of microbial features drives cytokine responses.

The limitations of our study include the unavailability of measures of bacterial load (cfu) and TTP; different durations of antibiotic treatment in the 1–3 days before serum was collected would variably reduce bacterial load. Cohorts with consistent early sampling are needed to assess whether bacterial DNA values are more predictive of adverse outcome when levels are not potentially confounded by antibiotics. Viable bacteria were excluded from our assay of cell-free DNA by centrifugation. Larger sample volumes are needed to determine whether the lack of detection of SAcfDNA in 45% of patients in this cohort was due to the small volume of serum available for this study or the timing of collection relative to antibiotic treatment. The case–control cohort was designed to study prognostic associations with persistent bacteremia and mortality and was underpowered for other disease outcomes such as recurrence. Validation in larger cohorts would increase confidence in our findings.

We propose that the clinical utility of a relatively simple-to-implement quantitative PCR assay measuring *S. aureus* cell-free DNA should be further explored. For example, high circulating bacterial cfDNA could identify patients with increased risk of endocarditis, to guide additional diagnostic imaging. Early SAcfDNA levels were superior to APACHE II score for mortality risk stratification, which could be clinically actionable as median time to death was approximately 3 weeks in this cohort. Using this PCR assay in a separate cohort with longitudinal sampling, Gutierrez et al. reported sustained bacterial DNA in patients with *S. aureus* bacteremia following clearance of blood cultures, with longer durations observed in complicated endocarditis and osteomyelitis [26]. Standard of care therapy requires 6–8 weeks of antibiotic treatment for patients with complicated endocarditis and osteomyelitis, long after blood cultures are negative in most patients. These 2 studies provide preliminary evidence for the possible utility of bacterial DNA for guiding antibiotic treatment length and monitoring response to therapy. This cohort represents a single center, and our findings need to be further evaluated in a validation cohort to understand if SAcfDNA could indicate the presence of recalcitrant infection with sufficient sensitivity and specificity to guide aggressive strategies for both diagnosis and treatment.

## Supplementary Data

Supplementary materials are available at *Open Forum Infectious Diseases* online. Consisting of data provided by the authors to benefit the reader, the posted materials are not copyedited and are the sole responsibility of the authors, so questions or comments should be addressed to the corresponding author.

Supplementary_Material
