# Supplementary material for: Prognostic Power of Pathogen Cell-Free DNA in Staphylococcus aureus Bacteremia
Source: Open Forum Infect Dis. 2019 Mar 15;6(4):ofz126. doi: 10.1093/ofid/ofz126 (PMC6483138; doi:10.1093/ofid/ofz126)
Supplement: Supplementary_Material [file ofz126_suppl_supplementary_material.pdf]

## SUPPLEMENTARY INFORMATION

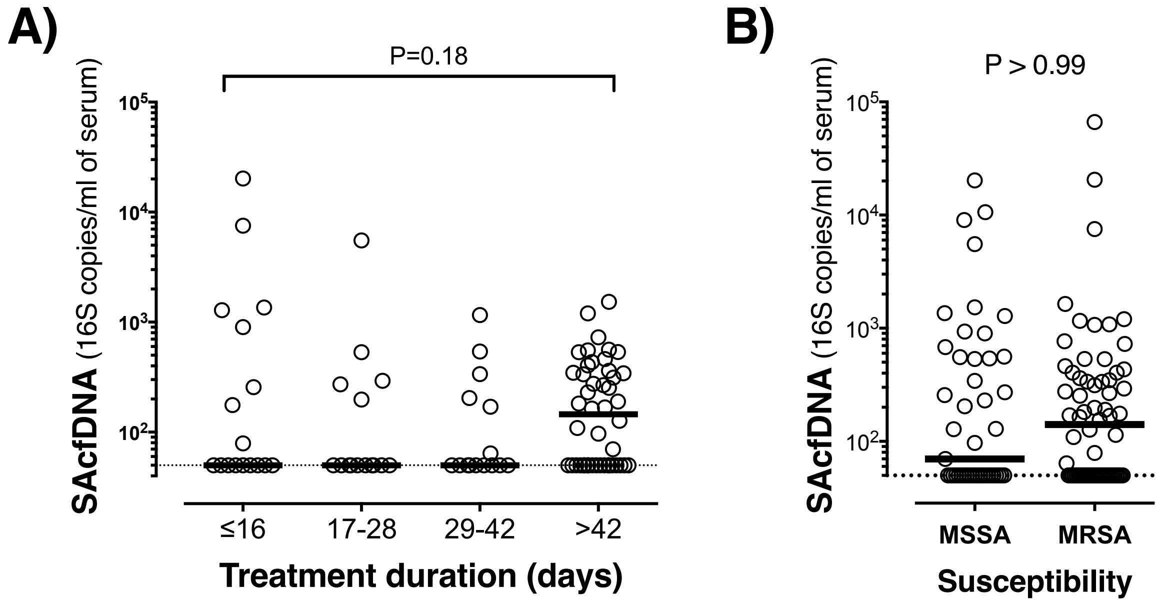


**Supplementary Figure 1**. Elevated bacterial cell-free DNA is not associated with treatment duration or methicillin resistant strain. **(A)** SA-cfDNA levels by days of antibiotic treatment (<17 d n=17, 17-28 d n=15, 29-42 d n=15, >42 d n=46). **(B)** SA-cfDNA levels by whether S. aureus is susceptible (MSSA, n=45) or resistant (MRSA, n=66) to methicillin. Medians and Wilcoxon rank sum test p values are shown, dotted lines indicates the assay LLOQ.

**Supplementary Table 1. Prognostic value of baseline biomarkers and clinical metrics for mortality, attributable mortality and persistent bacteremia.**

| | Mortality | | | Infection-related mortality | | | Persistent Bacteremia | | |
|---|---|---|---|---|---|---|---|---|---|
| | P value | | AUROC | P value | | AUROC | P value | | AUROC |
| IL-8 | <0.0001 | **** | 0.84 | <0.0001 | **** | 0.84 | 0.69 | | |
| CCL2 | <0.0001 | **** | 0.80 | <0.0001 | **** | 0.83 | 0.36 | | |
| IL-6 | <0.0001 | **** | 0.78 | <0.0001 | **** | 0.81 | 0.015 | * | 0.63 |
| IL-1RN | <0.0001 | **** | 0.75 | <0.0001 | **** | 0.80 | 0.0051 | ** | 0.65 |
| CHI3L1 | 0.0015 | ** | 0.71 | <0.0001 | **** | 0.78 | 0.087 | | |
| IL-10 | 0.0031 | ** | 0.69 | 0.0059 | ** | 0.70 | 0.00040 | *** | 0.69 |
| ANGPT2 | 0.0039 | ** | 0.69 | 0.0024 | ** | 0.72 | 0.23 | | |
| SAcfDNA | 0.0055 | ** | 0.67 | 0.00070 | *** | 0.73 | 0.0045 | ** | 0.65 |
| Age | 0.0075 | ** | 0.67 | 0.032 | * | 0.66 | 0.52 | | |
| CCL26 | 0.015 | * | 0.66 | 0.063 | | | 0.74 | | |
| G-CSF | 0.016 | * | 0.64 | 0.0016 | ** | 0.70 | 0.94 | | |
| IL-27 | 0.030 | * | 0.64 | 0.0035 | ** | 0.71 | 0.068 | | |
| APACHE II | 0.039 | * | 0.64 | 0.0082 | ** | 0.69 | 0.22 | | |
| CXCL10 | 0.043 | * | 0.63 | 0.072 | | | 0.79 | | |
| sTNFR1 | 0.10 | | | 0.048 | * | 0.65 | 0.15 | | |
| TREM1 | 0.22 | | | 0.048 | * | 0.65 | 0.12 | | |
| IL-17A | 0.42 | | | 0.95 | | | <0.0001 | **** | 0.73 |
| sE-Selectin | 0.25 | | | 0.91 | | | 0.0022 | ** | 0.67 |
| sIL-2RA | 0.36 | | | 0.17 | | | 0.018 | * | 0.63 |
| LCN2 | 0.18 | | | 0.057 | | | 0.032 | * | 0.62 |
| CCL5 | 0.72 | | | 0.27 | | | 0.036 | * | 0.62 |
| TNF | 0.068 | | | 0.068 | | | 0.38 | | |
| WBC | 0.11 | | | 0.085 | | | 0.15 | | |
| Hematocrit | 0.12 | | | 0.22 | | | 0.86 | | |
| BUN | 0.17 | | | 0.18 | | | 0.38 | | |
| Bands % | 0.18 | | | 0.16 | | | 1.00 | | |
| Creatinine | 0.19 | | | 0.34 | | | 0.66 | | |
| Platelets | 0.49 | | | 0.17 | | | 0.41 | | |
| Neutrophil % | 0.52 | | | 0.56 | | | 0.28 | | |

WBC=white blood cell counts; Platelets=platelet blood counts; BUN=blood urea nitrogen; AUROC is area under the receiver operating characteristic curves; P values from Wilcoxon rank sum tests are shown (* P < 0.05, ** P < 0.01 , *** P < 0.001, **** P < 0.0001).

**Supplementary Table 2. Correlation between baseline biomarkers and bacteremia duration or baseline serum level of *S. aureus* cell-free DNA.**

**Bloodstream infection duration**

| | Spearman ρ | | P value |
|---|---|---|---|
| IL-17A | 0.50 | **** | <0.0001 |
| IL-10 | 0.41 | **** | <0.0001 |
| SAcfDNA | 0.32 | *** | 0.0005 |
| sE-Selectin | 0.32 | *** | 0.0006 |
| sIL-2RA | 0.30 | ** | 0.0012 |
| LCN2 | 0.30 | ** | 0.0015 |
| IL-1RN | 0.29 | ** | 0.0019 |
| sTNFR1 | 0.29 | ** | 0.0022 |
| IL-6 | 0.28 | ** | 0.0026 |
| CHI3L1 | 0.28 | ** | 0.00 |
| TREM1 | 0.24 | * | 0.012 |
| TNF | 0.22 | * | 0.02 |
| IL-27 | 0.21 | * | 0.027 |
| APACHE II | 0.20 | * | 0.04 |
| ANGPT2 | 0.19 | * | 0.04 |
| BUN | 0.18 | | 0.06 |
| Creatinine | 0.17 | | 0.08 |
| CCL2 | 0.16 | | 0.10 |
| CXCL10 | 0.14 | | 0.13 |
| Neutrophil % | 0.12 | | 0.37 |
| WBC | 0.11 | | 0.26 |
| G-CSF | 0.10 | | 0.29 |
| Age | 0.07 | | 0.44 |
| IL-8 | 0.07 | | 0.46 |
| Bands % | 0.03 | | 0.83 |
| Hematocrit | -0.04 | | 0.71 |
| Eotaxin3 | -0.05 | | 0.61 |
| Platelets | -0.12 | | 0.20 |
| CCL5 | -0.24 | * | 0.01 |

***S. aureus* cell-free DNA**

| | Spearman ρ | | P value |
|---|---|---|---|
| G-CSF | 0.40 | **** | <0.0001 |
| IL-6 | 0.36 | *** | 0.0001 |
| IL-17A | 0.33 | *** | 0.0004 |
| IL-27 | 0.33 | *** | 0.0004 |
| B.I.Duration | 0.32 | *** | 0.0005 |
| IL-1RN | 0.29 | ** | 0.0018 |
| CHI3L1 | 0.29 | ** | 0.0020 |
| IL-10 | 0.27 | ** | 0.0041 |
| IL-8 | 0.25 | ** | 0.0070 |
| Bands % | 0.22 | | 0.13 |
| CCL2 | 0.20 | * | 0.035 |
| CCL26 | 0.19 | * | 0.04 |
| sE-Selectin | 0.17 | | 0.084 |
| ANGPT2 | 0.15 | | 0.11 |
| sIL-2RA | 0.14 | | 0.15 |
| LCN2 | 0.12 | | 0.19 |
| CXCL10 | 0.11 | | 0.24 |
| Age | 0.11 | | 0.27 |
| APACHE II | 0.09 | | 0.36 |
| BUN | 0.07 | | 0.47 |
| TNF | 0.07 | | 0.49 |
| TREM1 | 0.05 | | 0.59 |
| sTNFR1 | 0.05 | | 0.59 |
| WBC | -0.07 | | 0.46 |
| Creatinine | -0.09 | | 0.36 |
| Neutrophil % | -0.11 | | 0.44 |
| Platelets | -0.13 | | 0.19 |
| Hematocrit | -0.15 | | 0.13 |
| CCL5 | -0.16 | | 0.10 |

B.I.= bloodstream infection, WBC=white blood cell counts, Platelets=platelet blood counts; BUN=blood urea nitrogen, SAcfDNA= *S. aureus* cell-free DNA; Spearman rho and Wilcoxon rank sum P values are shown (* P < 0.05, ** P < 0.01 , *** P < 0.001).

**Supplementary Table 3. The contribution of SAcfDNA to logistic regression models including cytokines for mortality, attributable mortality and persistent bacteremia.**

| | All Cause Mortality | | Attributable Mortality | | Persistent Bacteremia | |
|---|---|---|---|---|---|---|
| | **Coefficients** | **P-value** | **Coefficients** | **P-value** | **Coefficients** | **P-value** |
| **Age** | 0.03 | 0.14 | 0.03 | 0.3 | 0.013 | 0.4 |
| **SAcfDNA** | 0.22 | 0.6 | 0.46 | 0.3 | 0.63 | 0.14 |
| **IL-8** | 2.58 | 0.005 ** | 2.24 | 0.01* | NA | NA |
| **CCL-2** | 4.95 | 0.01 * | 6.34 | 0.004 ** | NA | NA |
| **IL-17A** | NA | NA | NA | NA | 1.46 | 0.0005 *** |

P-values for the logistic regression coefficients are shown (* $P < 0.05$, ** $P < 0.01$ , *** $P < 0.001$).

## Supplementary Materials and Methods

*S. aureus cell-free DNA quantitative PCR*

Genomic DNA from *S. aureus* USA300 (FPR3757) was extracted and used as the standard. The mass of the genomic DNA and concentration was used to calculate 16S copy number for the standard curve. Quantitative PCR was carried out on the purified sample DNA and known copy number of DNA standard. Briefly, 20 µl reaction mixtures containing 5 µl DNA, 10 µl 2x TaqMan Fast Advanced master mix (Thermo Fisher Scientific), 1 µl 20x TaqMan primer/probe mix (Thermo Fisher Scientific), 4 µl nuclease-free water were prepared and subjected to real-time PCR with a QuantStudio 7 instrument (Thermo Fisher Scientific). The thermal cycling conditions were 50°C for 2 minutes and 95°C for 20 seconds, followed by 40 cycles of 95°C for 1 second and 60°C for 20 seconds. Samples were run in triplicate and mean cycle threshold (Ct) values were applied to the standard curve generated in the same experiment to obtain corresponding copy number of bacteria then converted to copy number per ml. The qPCR assay has been qualified with lower limit of quantitation (LLOQ) and upper limit of quantitation (ULOQ) of 5 and 107 copies,

respectively. For the limit of detection (LOD), a cycle threshold cut-off of 38 was set based on qualification experiments. The 16S rRNA gene copy number can vary from 5-6 copies per bacterium so measurements reflect total genome copies rather than an absolute measurement of bacterial load. All PCR data and LODs were normalized by blood or serum volume input into the PCR reaction and represented graphically as copies of 16S gene per mL of clinical sample.
